# Anxiety and depressive symptoms in women with fear of birth: A longitudinal cohort study

**DOI:** 10.18332/ejm/138941

**Published:** 2021-08-02

**Authors:** Ingegerd Hildingsson, Johanna Nilsson, Elida Merio, Birgitta Larsson

**Affiliations:** 1 Department of Women’s and Children’s Health, Uppsala University, Uppsala, Sweden; 2 Department of Nursing, Mid Sweden University, Sundsvall, Sweden; 3 Department of Health Promoting Science, Sophiahemmet University College, Stockholm, Sweden

**Keywords:** anxiety, depression, fear of birth, pregnancy, prenatal attachment, women

## Abstract

**INTRODUCTION:**

Anxiety and depression during pregnancy could imply difficulties in the attachment to the unborn baby. The objective of this study was to investigate the prevalence and change in anxiety and depressive symptoms in pregnant women with fear of birth. Another aim was to explore associations between symptoms of anxiety and depression on prenatal attachment.

**METHODS:**

This is a longitudinal cohort study of 77 pregnant women with fear of birth in three hospitals in Sweden. Data were collected by three questionnaires in mid and late pregnancy and two months after birth.

**RESULTS:**

Anxiety symptoms were more often reported than depressive symptoms, significantly decreasing over time in both conditions. Anxiety symptoms were associated with low education level, negative feelings towards the upcoming birth, and levels of fear of birth. Depressive symptoms were associated with levels of fear of birth. One in five women presented with fear of birth, anxiety, and depressive symptoms, suggesting that co-morbidity was quite common in this sample. Depressive symptoms and co-morbidity were negatively associated with prenatal attachment.

**CONCLUSIONS:**

This study shows that symptoms of anxiety and depression in women with fear of birth vary over time and that co-morbidity is quite common. Lack of emotional well-being was related to prenatal attachment. Healthcare professionals must identify and support women with anxiety and depressive symptoms and fear of birth so that difficulties in the relationship between the mother and the newborn baby might be reduced.

## INTRODUCTION

In recent years, more attention has been devoted to women’s emotional well-being during the perinatal period. Fear of birth, anxiety, and depressive symptoms are sometimes interrelated, but such a co-morbidity is not always acknowledged in healthcare services. Co-morbidity refers to people who have a disease or condition also have one or more other diseases or conditions.

The concept of fear of birth was first described in the 1980s, with a notion that some pregnant women had severe anxiety about birth that impaired daily life and well-being^1^. There is currently no uniform definition of fear of birth, and different measurement scales are used to identify women with such conditions, which makes it difficult to compare studies. Fear of birth can be categorized from mild to phobic, according to the Swedish Association of Obstetricians and Gynecologists^2^. It is often described as a primary fear of birth, which occurs in women who have not previously given birth, and secondary in women who have given birth. The latter can sometimes be the result of a previous traumatic birth^2^. The prevalence of fear of birth is currently around 12% in Scandinavian countries, as reported in a review article of 30 studies from 18 countries^3^. A multi-country European study showed a lower prevalence of fear in women with planned pregnancies and in women cohabiting with a partner. Women with poor finances who lack a social network were more likely to report fear of birth^4^. Another recent systematic review of 21 scientific papers focused on causes and outcomes of fear of birth. Stress, anxiety, and depressive symptoms together with lack of social support were associated with fear of birth^5^. Similar levels of fear were found in primiparous as well as multiparous women but for different reasons. Labor outcomes were also affected by fear; prolonged labor, epidural anesthesia, and obstetric complications were more prevalent in women with fear of birth. The strongest prediction for fear in multiparous women was a previous negative birth experience^5^.

There is a growing body of studies focusing on co-morbidity between depressive symptoms, anxiety and fear of birth. In a Norwegian study where 1642 women responded to a questionnaire in gestational week 32, 8.9% of the women presented with symptoms of depression and 8.8% with anxiety symptoms. A total of 32% reported depression and fear of birth and 12% anxiety and fear of birth. Women who had symptoms of both depression and anxiety were also more likely to suffer from fear of birth^6^. Similarly, a study from Finland reported a three-fold increased risk for women with fear of birth to suffer from severe depression^7^, which also was confirmed in a Finnish register study where anxiety and depression were twice as prevalent in women with fear of birth^8^.

It has previously been shown that depressive symptoms and anxiety during pregnancy can have a negative impact on fetal development. Studies have shown an increased risk of pre-term birth, low Apgar scores, stillbirths, and congenital malformations in babies born to women with severe depression^7^. It is also known that fear of birth can adversely affect a woman’s ability to relate to the newborn baby^9^ and that fear may be linked to the occurrence of postpartum depression in the mother^1^. Anxiety during pregnancy is also associated with an increased risk of postpartum depression^10^. Furthermore, prenatal anxiety could result in negative consequences for the baby, such as delayed social, emotional, and cognitive development^11^. Women with prenatal anxiety are also less likely to breastfeed their babies^10^.

Prenatal attachment is a concept defined as the unique relationship between the mother-to-be and her unborn baby^12^, important for the adjustment to the pregnancy in terms of health behaviors. Prenatal attachment activities could be that the mother-to-be has fantasies about the baby, its personality and activities, recognizes her feelings for the baby and interacts with the unborn baby^12^. Several instruments have been developed to measure prenatal attachment, one is the Prenatal Attachment Inventory^12^. Lower levels of prenatal attachment have been found in younger women^13,14^, in women being pregnant after *in vitro* fertilization^15^ and in women who prefer a cesarean section or have negative feelings towards the coming birth^14^. Previous studies have also identified that women with anxiety or depressive symptoms are more likely to have altered levels of prenatal attachment^14,16^. In a population-based study of 718 women^14^, the results showed that women with depressive symptoms had lower levels of attachment on the three subscales of the revised version of the Prenatal Attachment Inventory (PAI-R)^17^, while women with elevated levels of anxiety scored higher on one of the subscales, i.e. Anticipation^14^.

Previous research has shown that many women experience fear of birth, and that there is a link between fear of birth and impaired emotional well-being. Prevalence studies are fairly common, but few studies have focused on how symptoms of anxiety and depression change during the childbearing period, especially in women with fear of birth. Currently, there is a growing awareness of prenatal mental health problems and their consequences for the woman and the baby. Persistent depressive symptoms could have a huge impact for the new-born baby and the child’s development. In Sweden, the majority of screening procedures takes place after birth. Identifying women already during pregnancy would give healthcare professionals increased opportunities to timely refer women to treatment.

The aim of this study was to investigate the prevalence and change in anxiety and depressive symptoms in pregnant women with fear of birth. An additional aim was to explore associations between symptoms of anxiety and depression on prenatal attachment.

## METHODS

### Study design

This is part of a prospective longitudinal cohort study of women referred to counselling for their fear of birth. Details of the study are reported elsewhere^18^. In short, women referred to counselling were offered to have their intrapartum care from a counselling midwife whom they previously met, if possible. The 13 midwives working with counselling were experienced in intrapartum care and were often in charge of the labor ward as team coordinators. They were not on-call to provide continuity of care for the referred women, but if they were present the day the women went into labor, they did their best to change their working situation and assisted during labor. The prevalence of a known midwife for the women was 34%^18^.

### Setting

Three hospitals in the middle and northern part of Sweden, with annual birth rates of 1525, 1329 and 1564, respectively, were chosen. The hospitals provided midwifery-led counselling for around 150 women each year. Women were referred to the counselling team by the antenatal midwife if they self-reported fear of birth. Usually, the referral was made after the 24th gestational week.

### Participants

All women who were referred to counselling for fear of birth in the three hospitals were asked to participate in the study, and 77 women consented. The main reason for not participating was having a planned caesarean section. The women had an estimated date of birth from 1 September 2016 to 31 May 2017. To participate in the study, the requirement was to have sufficient knowledge of Swedish or English to be able to complete the questionnaires^19^.

### Data collection

Data were collected by three questionnaires: in mid and late pregnancy and two months after the birth. After receiving consent, the questionnaires were sent to the women’s home address together with a pre-paid return envelope. The first questionnaire included background questions (age, marital status, country of birth, education level, parity, previous mental illness, previous birth experience, birth attitudes and preferences). Fear of birth was assessed using the Fear of Birth Scale (FOBS). The FOBS scale consists of two 100 mm Visual Analog Scales that are summed and averaged to get a score. When filling in the scale, study participants are asked to respond to the question ‘How do you feel right now about the approaching birth?’ and are instructed to place a mark on the two scales which have the anchor words calm/worried and no fear/strong fear. A FOBS score ≥60 was used to classify women with fear of birth^20,21^. FOBS has been psychometrically tested and validated against the Wijma Delivery Expectancy Questionnaire (WDEQ), in a large Australian sample^22^. The correlation between the instruments was strong (Rho=0.66, p<0.001). The area under the ROC was 0.89 indicating high sensitivity with a FOBS cut-off point of 54. Sensitivity was 89%, specificity 79% and Youden index 0.68. Positive predictive value was 85% and negative predictive value 79%. For practical reasons, after discussion with counseling midwives and obstetricians a FOBS score ≥60 is now used in clinical practice.

The Hospital Anxiety and Depression Scale (HADS) was used to investigate the women’s emotional well-being^23^. The HADS measures both anxiety and depressive symptoms (HADS-A vs HADS-D). The instrument is intended to help healthcare providers obtain an early indication of impaired emotional well-being. The scale has been validated, and it is useful and easy to administer; however, it is not a diagnostic tool^24,25^. Each question has four response options ranging from 0 to 3, where 0 means ‘no symptoms’ and 3 ‘severe symptoms.’ The creators of the scale have proposed 0–7 for normal intervals, 8–10 as a cause of concern, and ≥11 as a possible clinical case requiring assessment^23^. In the present study, ≥8 was chosen as indicating symptoms of anxiety and depression. The HADS was repeated in the questionnaires in late pregnancy and two months after birth.

From the questionnaire completed in late pregnancy, prenatal attachment was identified, using the revised version of the Prenatal Attachment Inventory^17^. In the revised version, which builds on a population-based sample of pregnant women, three subscales were identified after explanatory and confirmatory factor analysis and Rash analysis. The three subscales were labelled: Anticipation, Interaction, and Differentiation. The subscale Anticipation includes items about fantasies and future plans for the baby. The subscale Interaction includes items that mirror the woman’s feelings for her baby and sharing her experience with others. The subscale Differentiation refers to knowledge about the baby’s personality and activities. Higher mean scores indicate higher levels of attachment. The Cronbach alpha value for the total scale was 0.859 in the present sample.

### Statistical analysis

The software SPSS version 24 was used in the analysis. Some of the background data were dichotomized to facilitate further analysis. Age was dichotomized into <33 years and ≥33 years, and country of birth into Sweden versus other countries. Education level was dichotomized into low (compulsory school + high school) and high (college + university). The women were also asked about previous mental health issues, such as depression, anxiety, eating disorders, bipolarity, and neuropsychiatric disabilities. From this information, a new variable was created and named ‘previous mental illness’ versus ‘no previous mental illness’. Previous birth experience was assessed on a 5-point rating scale ranging: 1=‘Very positive’ to 5=‘Very Negative’. It was dichotomized into ‘Positive birth experience’ and ‘Less than positive birth experience’ (1=Positive and Very positive, 0=Mixed feelings, Negative, and Very negative). A question about feelings about the forthcoming birth was initially dichotomized in a similar way, but due to the small number of women reporting positive feelings about the forthcoming birth, it was decided to dichotomize the variable into positive + mixed feelings=0 and negative feelings=1.

Descriptive statistics were used to present the population under study. To study changes in HADS scores over time, repeated measures analysis of variance was performed separately for HADS-A and HADS-D, with the effect size measured with partial n^2^ and the guidelines proposed by Cohen 0.01=small effect, 0.06=moderate effect, and 0.13=large effect. The total sum of HADS-A and HADS-D was dichotomized into 0–7 and ≥8 (indicating symptoms of anxiety or depression). Odds ratios with a 95% confidence interval were calculated between women with and without symptoms of anxiety and depression, in relation to the exploratory background variables. Mean scores were calculated between the dichotomized HADS-A and HADS-D and the subscales of the Prenatal Attachment Inventory, using t-tests. A composite variable was also created to investigate the prevalence of co-morbidity. In that variable, women with anxiety and depressive symptoms scores were grouped together with women presenting FOBS ≥60.

The study was approved by the Regional Ethics Committee (Date/number: 2016/0588).

## RESULTS

The sample included 77 pregnant women, all of whom completed the questionnaire in mid pregnancy. Table 1 shows the women’s sociodemographic and obstetric backgrounds. The mean age was 32.55 years, and the majority lived with a partner and were born in Sweden. Around 60% had a college or university level of education, and nearly half had some previous mental illness. The majority had previous children (74%), and more than half had a negative birth experience during the most recent birth (58%). When thinking about the forthcoming birth, the majority (66%) reported mixed feelings, and one in three women had negative feelings about the approaching birth. One-third of the women stated that, if they had the opportunity to choose, they would prefer to have a caesarean section.

**Table 1:** Background characteristics of the participants (N=77)

Characteristics	*n* (%)
**Age** (years)	
<33	40 (51.9)
≥33	37 (48.1)
Mean ± SD	32.55 ± 5.06
**Marital status**	
Living with partner	76 (98.7)
Not living with partner	1 (1.3)
**Country of birth**	
Sweden	70 (90.9)
Other	7 (9.1)
**Education level**	
Compulsory school/high school	31 (40.3)
College/university	46 (59.7)
**Previous mental illness**	
None	40 (51.9)
Any mental illness	37 (48.1)
**Parity**	
Primiparas	20 (26.0)
Multiparas	57 (74.0)
**Obstetric history**	
Previous miscarriage	22 (28.9)
Previous abortion	21 (27.6)
Infertility problems	4 (5.3)
**Previous birth experience**	
Positive or mixed feelings	24 (42.1)
Negative	33 (57.9)
**Feelings when thinking about the approaching birth**	
Positive	3 (3.9)
Mixed	50 (65.8)
Negative	23 (30.3)
**Birth preference**	
Vaginal	52 (67.5)
Caesarean section	25 (32.5)
**Fear of birth scale**	
FOBS (mean ± SD)	72.53 ± 21.25
FOBS <60	17 (22.1)
FOBS ≥60	60 (77.9)

Table 2 shows the change in the mean scores of HADS over time in 61 women who completed all three measures of HADS. There was a statistically significant difference in the HADS over time, for anxiety as well as depressive symptoms. For anxiety symptoms, the mean value was highest in mid pregnancy, decreased in late pregnancy, and further diminished two months after birth. Depressive symptoms showed a slightly different pattern, with a moderate mean score of 5.09 in mid pregnancy, an increase in late pregnancy, and then a decrease two months after birth. The partial n^2^ values indicated that the effect sizes were large for all measures.

**Table 2:** Mean scores (SD) and change in the Hospital Anxiety and Depression (HADS) scores over time (Ν=61)

	*Mid pregnancy*	*Late pregnancy*	*After birth*	*p*	η^2^*
	*mean (SD)*	*mean (SD)*	*mean (SD)*		
Total HADS	12.63 (7.25)	13.09 (6.32)	8.19 (6.64)	0.000	0.355
HADS-anxiety	7.54 (4.81)	7.31 (3.77)	4.50 (3.60)	0.000	0.387
HADS-depression	5.09 (3.87)	5.78 (3.81)	3.68 (3.90)	0.002	0.198

* Repeated measures ANOVA.

### Prevalence of anxiety and depressive symptoms

The prevalence of anxiety (HADS-A score ≥8) in mid-pregnancy was 53.2%, and the prevalence of depressive symptoms (HADS-D score ≥8) was 28.6%. In late pregnancy, the corresponding prevalence was 51.6% for anxiety and 31.3% for depressive symptoms. Two months after birth, the prevalence of anxiety and depressive symptoms decreased to 21.1% and 11.3%, respectively (not shown).

### Background characteristics in relation to anxiety and depressive symptoms on three measures

In Table 3, symptoms of anxiety and depression are compared with the women’s background characteristics. Few sociodemographic background characteristics were associated with anxiety or depressive symptoms, with the exception of low education level being associated with anxiety. Negative feelings about the forthcoming birth, mean scores of FOBS, and FOBS >60 also increased the risk of anxiety. HADS-D score >8 was associated with mean scores of FOBS and FOBS scores >60.

**Table 3:** Symptoms of anxiety and depression in relation to background characteristics in mid pregnancy using the HADS

*Variable*	*HADS-anxiety*				*HADS-depression*			
	*<8*	*≥8*			*<8*	*≥8*		
	*(n=36)*	*(n=41)*		*p*	*(n=55)*	*(n=22)*		*p*
	*n (%)*	*n (%)*	*OR (95% CI)*		*n (%)*	*n (%)*	*OR (95% CI)*	
**Age** (years)								
<33 (Ref.)	16 (44.4)	24 (58.5)	1		25 (45.5)	15 (68.2)	1	
≥33	20 (55.6)	17 (41.5)	0.95 (0.87–1.03)	0.257	30 (54.5)	7 (31.8)	0.91 (0.83–1.00)	0.830
**Country of birth**								
Sweden (Ref.)	33 (91.7)	37 (90.2)	1		50 (90.9)	20 (90.9)	1	
Other	3 (8.3)	4 (9.8)	0.84 (0.17–4.03)	1.000	5 (9.1)	2 (9.1)	1.00 (0.17–5.58)	1.000
**Education level**								
Compulsory school/high school	10 (27.8)	21 (51.2)	2.73 (1.05–7.07)		19 (34.5)	12 (54.5)	2.27 (0.83–6.22)	
College/university (Ref.)	26 (72.2)	20 (48.8)	1	0.039	36 (65.5)	10 (45.5)	1	0.119
**Previous mental illness**								
None (Ref.)	23 (63.9)	17 (41.5)	1		32 (58.2)	8 (36.4)	1	
Any mental illness	13 (36.1)	24 (58.5)	2.49 (0.99–6.27)	0.051	23 (41.8)	14 (63.6)	2.43 (0.87–6.75)	0.087
**Parity**								
Primiparas (Ref.)	9 (25.0)	11 (26.8)	1		13 (23.6)	7 (31.8)	1	
Multiparas	27 (75.0)	30 (73.2)	0.90 (0.32–2.52)	1.000	42 (76.4)	15 (68.2)	0.66 (0.22–1.97)	0.567
**Previous birth experience** (multiparas)								
Positive/mixed (Ref.)	13 (48.1)	10 (34.5)	1		21 (50.0)	3 (20.0)	1	
Negative	14 (51.9)	19 (65.5)	1.60 (0.55–4.62)	0.429	21 (50.0)	12 (80.0)	4.00 (0.98–16.25)	0.053
**Previous birth experience**								
Positive/mixed (Ref.)	30 (83.3)	23 (57.5)	1		39 (72.2)	14 (63.6)	1	
Negative	6 (16.7)	17 (42.5)	3.69 (1.25–10.85)*	0.017	15 (27.8)	8 (36.4)	1.48 (0.52–4.25)	0.461
**Birth preference**								
Vaginal (Ref.)	26 (72.2)	26 (62.4)	1		38 (69.1)	14 (63.6)	1	
Caesarean section	10 (27.8)	15 (36.6)	1.50 (0.57–3.94)	0.470	17 (30.9)	8 (36.4)	1.27 (0.45–3.61)	0.788
**Fear of birth scale**								
FOBS (mean±SD)	63.09±23.20	80.81±15.38		0.000	68.59±21.74	82.38±16.63		0.009
<60 (Ref.)	14 (38.9)	3 (7.3)	1		15 (27.3)	2 (9.1)	1	
≥60	22 (61.1)	38 (92.7)	8.06 (2.08–31.1)	0.002	40 (72.7)	20 (90.9)	3.75 (0.78–18.02)	0.128

The same explanatory variables were also checked against having symptoms in late pregnancy and two months after birth. In late pregnancy, low education level (OR=5.65; 95% CI: 1.83–17.45), previous mental illness (OR=3.23; 95% CI: 1.15–9.02), and FOBS >60 in early pregnancy (OR=9.78; 95% CI: 1.97–48.59) were associated with anxiety symptoms in late pregnancy. Previous mental illness was also related to depressive symptoms (OR=2.95; 95% CI: 1.0–8.57). Two months after birth, symptoms of anxiety were associated with having had negative feelings about the forthcoming birth in mid pregnancy (OR=4.77; 95% CI: 1.38–16.45). The latter was also associated with depressive symptoms two months after birth (OR=10.07; 95% CI: 1.82–55.57).

To further understand the co-morbidity of anxiety, depression, and fear of birth, a composite variable showed that 20.3% of the sample suffered from all three conditions in mid pregnancy. The background variables associated with such a co-morbidity were being aged <33 years (OR=5.31; 95% CI: 1.52–18.49), low education level (OR=3.17; 95% CI: 1.01–9.94), and previous mental illness (OR=4.32; 95% CI: 1.24–14.98).

### The impact of anxiety and depressive symptoms on prenatal attachment

Having anxiety symptoms in mid pregnancy was not associated with any of the dimensions on the Prenatal Attachment Inventory. Women who presented with depressive symptoms in mid pregnancy were more likely to score lower on the dimension Interaction (p=0.037) compared to women without depressive symptoms. This subscale refers to women’s feeling for the baby and sharing experiences with others. Women with co-morbidity in mid pregnancy (fear of birth, anxiety, and depressive symptoms) scored lower on the dimension Differentiation (p=0.025). The subscale Differentiation refers to the woman’s knowledge about the baby’s personality and activities. No association was found between anxiety, depression or co-morbidity and the subscale Anticipation, which refers to fantasies and future plans for the baby.

**Figure 1:**
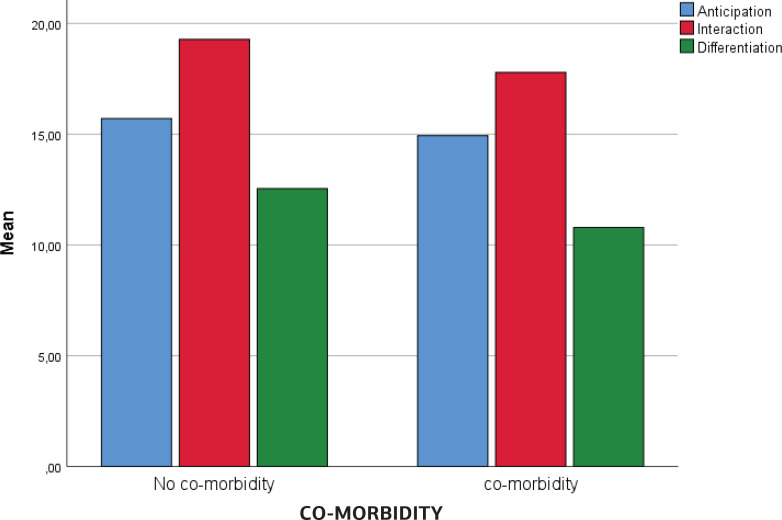
Co-morbidity in mid pregnancy in relation to the subscales of Prenatal Attachment Inventory-Revised

Figure 1 presents an overview of the levels of PAI-R subscales in mid pregnancy in women with and without co-morbidity. In late pregnancy, anxiety only, or depressive symptoms only, were not related to the PAI-R scores, but those with late co-morbidity were more likely to score lower on the dimension Differentiation (p=0.040). Only one background factor was associated with Attachment scores, as multiparous women scored higher in the subscale Differentiation compared to primiparous women (p<0.002).

## DISCUSSION

The main findings of this study were that anxiety and depressive symptoms were frequently reported in a sample of women referred to counselling for fear of birth and that there was a change in the levels of these symptoms over time. Co-morbidity was found in more than 20%. A few background variables were associated with anxiety symptoms but not with depressive symptoms. Depressive symptoms or co-morbidity were associated with lower levels of prenatal attachment on two subscales, Interaction and Differentiation.

### Prevalence of anxiety and depressive symptoms over time

The prevalence of anxiety in the present study was higher than the prevalence of depressive symptoms. Similar findings have previously been reported from a population-based Swedish study^26^. More than half of the women in the study presented with anxiety symptoms in mid and late pregnancy, but the prevalence of anxiety decreased two months after birth. Decrease in anxiety levels over the course of pregnancy has previously been reported^27,28^. The decrease in anxiety symptoms could be a result of the birth of a healthy baby^28^. Another explanation could be that anxiety and fear of birth are more closely related than fear of birth and depressive symptoms. Rondung et al.^29^ suggested a change in the wording of fear of birth to childbirth anxiety. This suggestion was based on a cluster analysis of several psychological measures from a randomized controlled trial of treatment for fear of birth. Interestingly, the correlation between psychological variables and fear of birth was low, and no single variable could predict the level of fear in the study.

Depressive symptoms also changed over time, with higher scores in late pregnancy compared to mid pregnancy and then again lower two months after birth. The prevalence reported two months after birth was quite similar to other studies of pregnant populations, 9–12%^30,31^.

Co-morbidity was also quite prominent in the present study, with 20% presenting fear of birth, anxiety, and depressive symptoms together. Similarly, a Norwegian population-based study of 1642 women in late pregnancy reported that 12% of women presented with anxiety symptoms and fear of birth, and depressive symptoms and fear of birth were found in one in three women; furthermore, women with both depressive symptoms and anxiety were more at risk for having fear of birth^6^. It is, therefore, important for healthcare providers to repeatedly ask women during and after pregnancy about symptoms of impaired emotional well-being. Some clinics in Sweden use a screening procedure to assess fear of birth, but the findings of the present study suggest that screening for mental health during pregnancy is also important. Currently, the Swedish Association of Obstetricians and Gynecologists recommends certain questions to be asked about emotional and mental health^32^. We do not know the extent of these recommendations from the current sample, or if women feel more stigmatized talking about mental health problems. Previous studies have reported that women could be ashamed of disseminating, for example, depressive symptoms^33^ during a period when one is supposed to be happy. In Sweden, the organization for counselling for fear of birth is available in all hospitals and has been a focus since the mid-1990s. It might, therefore, be easier to talk about fear of birth than mental health issues.

### Background factors associated with anxiety and depressive symptoms

Low education level was associated with anxiety symptoms, both in mid and late pregnancy and also with co-morbidity. Similar findings have also been reported by others^6,26,34^. A history of mental health problems was found in nearly half of the women in the present study, and such a history was related to having anxiety or depressive symptoms in late pregnancy and to the occurrence of suffering from anxiety, depressive symptoms and fear of birth together. This has also been reported in previous studies^6,35,36^. On the other hand, parity was not associated with any elevated levels of mental health issues in the present study and might be explained by the large proportion of multiparous women or the limited sample size in the present study. In contrast, previous studies have shown a lower prevalence of anxiety in multiparous women^11,34,37^.

### Prenatal attachment and associated factors

Prenatal attachment was also associated with women’s mental health, where women presenting with depression only or co-morbidity scored lower on the subscales Interaction and Differentiation. Similar findings have been previously been reported^24,38^. Flykt et al.^38^ suggested that prenatal depressive symptoms might have a higher influence than postnatal depressive symptoms when it comes to lack of responsiveness to the baby. Depressive symptoms might hinder the woman from interacting with the unborn baby and sharing her experiences with others and might also result in the woman losing interest in investigating the baby’s personality and activities.

Parity was the only background variable associated with prenatal attachment, showing that multiparous women were more likely to score higher than primiparous women on the subscale Differentiation. This is similar to a Swedish population-based study of 456 women in late pregnancy^39^. One explanation could be that women with previous children recognize fetal movements more often than first-time mothers.

The process of prenatal attachment between a pregnant woman and her unborn baby warrants further research and clinical attention. Healthcare providers who encounter pregnant women can easily raise women’s awareness of the baby’s characteristics by talking about the baby and asking about fetal movements and how these are experienced^40^. A perception of low attachment in pregnant women deserves further exploration of underlying emotional distress, such as anxiety or depressive symptoms.

### Strengths and limitations

This study has several limitations. The self-reported assessments, lack of psychiatric clinical diagnoses, and under-representation of foreign-born women in the study sample reduce its capacity to be generalized to wider populations of pregnant women. The fairly small sample size and its specific characteristics, i.e. fear of birth, further limit generalizability. Nevertheless, focusing on fear of birth adds to the growing body of women’s mental health issues. The study’s strength lies in the longitudinal design, making it available to follow the course of anxiety and depressive symptoms over time, in contrast to only measuring mental health on a single occasion^28^. Using validated scales to measure anxiety, depressive symptoms, fear of birth, and prenatal attachment, adds to the strength of the study.

## CONCLUSIONS

This study shows that symptoms of anxiety and depression in women with fear of birth varies over time and that co-morbidity is quite common. Anxiety and depressive symptoms could affect the prenatal attachment. It is, therefore, important that healthcare professionals identify and support women with anxiety and depressive symptoms in addition to fear of birth in order to avoid negative consequences like postpartum depression. Difficulties in the prenatal attachment between the mother and the baby might be reduced when focusing on these topics.

## Data Availability

The data supporting this study cannot be made available for privacy reasons.
